# Impact of machined versus structured implant shoulder designs on crestal bone level changes: a randomized, controlled, multicenter study

**DOI:** 10.1186/s40729-022-00432-4

**Published:** 2022-07-16

**Authors:** Daniel Rothamel, Maria Heinz, Daniel Ferrari, Alfons Eissing, Henrik Holtmann, Lara Schorn, Tim Fienitz

**Affiliations:** 1 Department of Oral-, Maxillofacial and Facial Plastic Surgery, University Hospital Cologne, Kerpener Str. 62, 50937 Cologne, Germany; 2 Department of Oral-, Maxillofacial and Facial Plastic Surgery, Evangelisches Krankenhaus Bethesda, Ludwig-Weber-Str. 15, 41061 Mönchengladbach, Germany; 3 Private Practice for Dentistry, Heinrichstraße 83-85, 40239 Düsseldorf, Germany; 4 Private Practice for Oral-, Maxillofacial and Facial Plastic Surgery, Pestalozzistraße 1B, 49808 Lingen, Germany; 5 Department of Oral-, Maxillofacial and Facial Plastic Surgery, University Hospital Duesseldorf, Moorenstr. 5, 40225 Düsseldorf, Germany

**Keywords:** Implant design, Machined vs. rough implant collar, Peri-implantitis, Peri-implant bone level

## Abstract

**Purpose:**

The collar region of an implant is its connection to the oral cavity. A balance between osseointegration on one hand and the absence of plaque accumulation on the other hand is necessary for successful implantation. It is yet to be determined which implant collar design, polished or rough, is best to stabilize the crestal bone level, avoiding peri-implantitis and subsequent risk of implant loss. The aim of this study was to investigate the influence of the architecture of the collar region on marginal bone and soft tissue response.

**Methods:**

This prospective, randomized, clinically controlled multicenter study included 58 patients undergoing dental implant treatment using a pair of dental implants with either machined or rough-surfaced shoulder regions. Patients were clinically and radiologically examined for bone level height and signs of inflammation after 6, 12 and 24 months.

**Results:**

No implant was lost within the 2 years of follow-up (100% survival rate). No significant differences on crestal bone loss (machined neck: 0.61 mm ± 0.28 mm, rough neck 0.58 mm ± 0.24 mm) and on soft tissue response (probing depth 3–6 mm with bleeding on probing 7.6% in machined-neck implants and in 8.3% in rough neck implants) were observed between implants with machined and roughened neck after 2 years.

**Conclusions:**

Machined and roughened neck implants achieved equally good results concerning peri-implant bone loss, the rate of peri-implantitis and implant survival rate/hard and soft tissue integration. None of the two collar designs showed a clear advantage in peri-implant reaction.

*Trial registration* German Clinical Trials Register, DKRS00029033. Registered 09 May 2022—Retrospectively registered, http://www.dkrs.de

**Supplementary Information:**

The online version contains supplementary material available at 10.1186/s40729-022-00432-4.

## Background

Within the last decades, dental implant treatment has become more and more indispensable in modern dentistry to restore missing teeth and preserve the bone from natural atrophy.

To improve osseointegration of dental implants, many different implant configurations have been designed over time. Among the current 1300 different implant systems there is little discussion about the general design of dental implants [1]. The macro-design describes the broad shape (conical or cylindrical), length, diameter, arrangement and number of threads and numerous other characteristics/configurations of a dental implant. Common implant shapes are cylindrical or tapered [2]. The micro-design describes the surface characterization of the implant. Design features of the implant–prosthesis–abutment complex, such as the cervical margin, emergence angle and emergence profile, as well as the design of the implant–abutment and abutment–prosthesis junctions and their locations in relation to the oral tissues, can significantly impact peri-implant tissues in the long term [3]. There is an ongoing discussion in literature whether the broad part of the implant, which is supposed to be covered by bone, should be designed in a more or less rough way in order to directly increase cell accumulation and therefore favor osseointegration [4]. The roughness of an implant’s surface is achieved either by reductive or additive procedures. The majority of implants hold moderately rough surfaces (Ra = roughness between 1.0 and 2.0/3.0 micron/µm) as these show stronger bone responses than smoother or rougher implant surfaces [4–6]. Unfortunately, the effect of cell accumulation on rough implant surfaces does not only favor bone cell adhesion. Rough implant surfaces increase cell accumulation in general, attracting fibrous tissue and bacteria and therefore favor peri-implant infections of the surrounding soft and hard tissue [7, 8]. A greater surface roughness comes with increased biofilm formation around the implant [9, 10]. A review including 22 articles showed that implant roughness is clearly associated with peri-implantitis. The higher the surface roughness, the higher the mean per-implantitis rate. Up to an arithmetic mean surface roughness of Ra 1 µm, a significant reduction of peri-implantitis was found. Higher risk of peri-implantitis appeared for Ra values greater than 1.2 µm [11]. Other studies revealed that surface roughness below a Ra value of 0.2 microns had no further influence on the quantity and composition of the biofilm formation, bacterial adhesion and colonization [12, 13]. Regarding this, Meier et al. recommended a surface roughness on implant parts in contact to the oral cavity below that value of 0.2 µm, as this value seems to indicate a threshold for increased biofilm accumulation [14].

In the collar region of implants, which is exposed to the oral cavity, it is crucial to avoid plaque accumulation. A balance between osseointegration on one hand and the absence of plaque accumulation on the other hand has been found to achieve optimal long-term clinical outcomes. Thus, it is to clarify the resulting question: which implant collar design is best to stabilize the crestal bone level, avoiding peri-implantitis and subsequent risk of implant loss? Until now, there is no consensus in literature favoring one over the other. Concerning the impact of the machined collar neck width, there is a tendency to keep the machined collar short, as implants with shorter machined collar regions appeared to be more effective in decreasing marginal bone loss [15, 16].

In the present study, BEGO Sedamos RS- and RSX-implants were used to directly compare the effect of machined versus rough-surfaced shoulders/collar regions on the surrounding tissue. Primarily, the peri-implant bone formation was evaluated, the secondary objective was to assess indicators for soft tissue inflammation.

## Methods

### Set-up

**Fig. 1:**
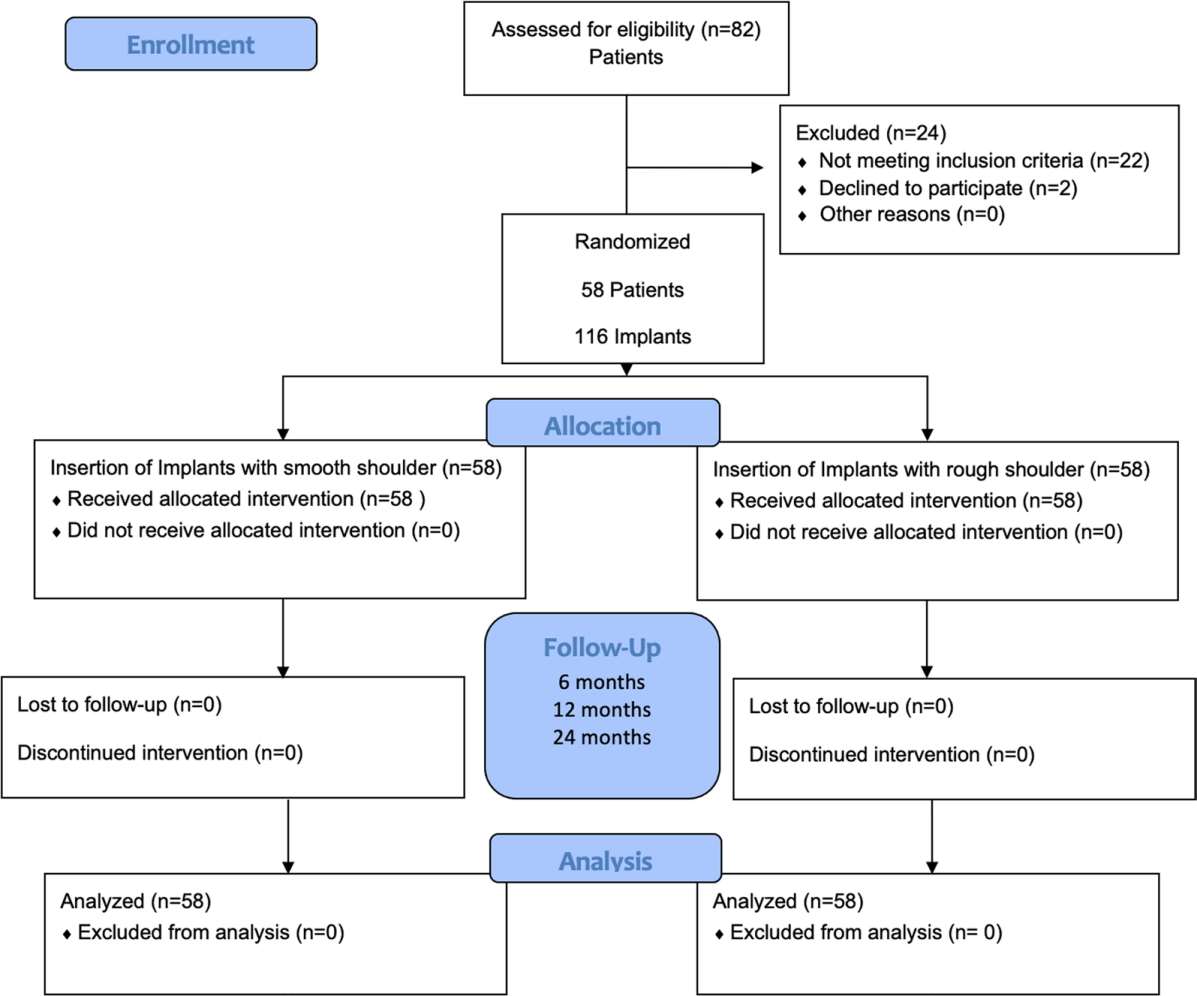
Study design including evaluated patients and implants (modified CONSORT flow diagram)

This prospective, randomized, clinically controlled multicenter study was performed within a time period of 24 months (Fig. 1). 58 patients underwent dental implant treatment with a pair of dental implants with slightly different designed shoulder regions. Apart from the shoulder regions, the implants shared exactly the same implant characteristics. Treatment, clinical follow-up examinations and radiographs were performed in three different centers (Department of Oral and Maxillofacial Surgery of the University of Cologne, Germany; Private dental practice Dr. Daniel Ferrari, Düsseldorf, Germany and Private dental practice Dr. Dr. Eißing, Lingen, Germany). The Study was approved by the ethics committee of the University of Cologne, Germany (University Cologne; 14-369). Sample size was based on a power analysis including an additional drop-out rate of 5% (a priori power analysis, Effect-size 0.5, G*Power, Heinrich Heine University, Düsseldorf, Germany). Written informed consent was provided by all patients before treatment. The study was conducted following the CONSORT checklist and was registered in the German Clinical Trials Register (DRKS00029033).

### Inclusion and exclusion criteria

Inclusion criteria for participating in the present study were: age between 18 and 85 years of age, indication for a dental implant treatment in two comparable regions of the jaw (opposite or next to each other), indication for fixed dental prosthetics (ceramic, single crown or dental bridge), written informed consent, and good oral hygiene.

Exclusion criteria were: not meeting inclusion criteria, consumption of cigarettes (more than 10 per day), existing periodontitis, uncontrolled systematic diseases with ASA 2 or more (ASA = American Society of Anesthesiologists), removable dentures, medication known to interfere in bone metabolism (antiresorptive medication), disease accompanied with disturbance in bone metabolism (Morbus Paget, osteoporosis, osteomalacia), conditions following extended operations or radiotherapy of the jaw, infection in or nearby operation sites.

### Insertion of implants

**Fig. 2:**
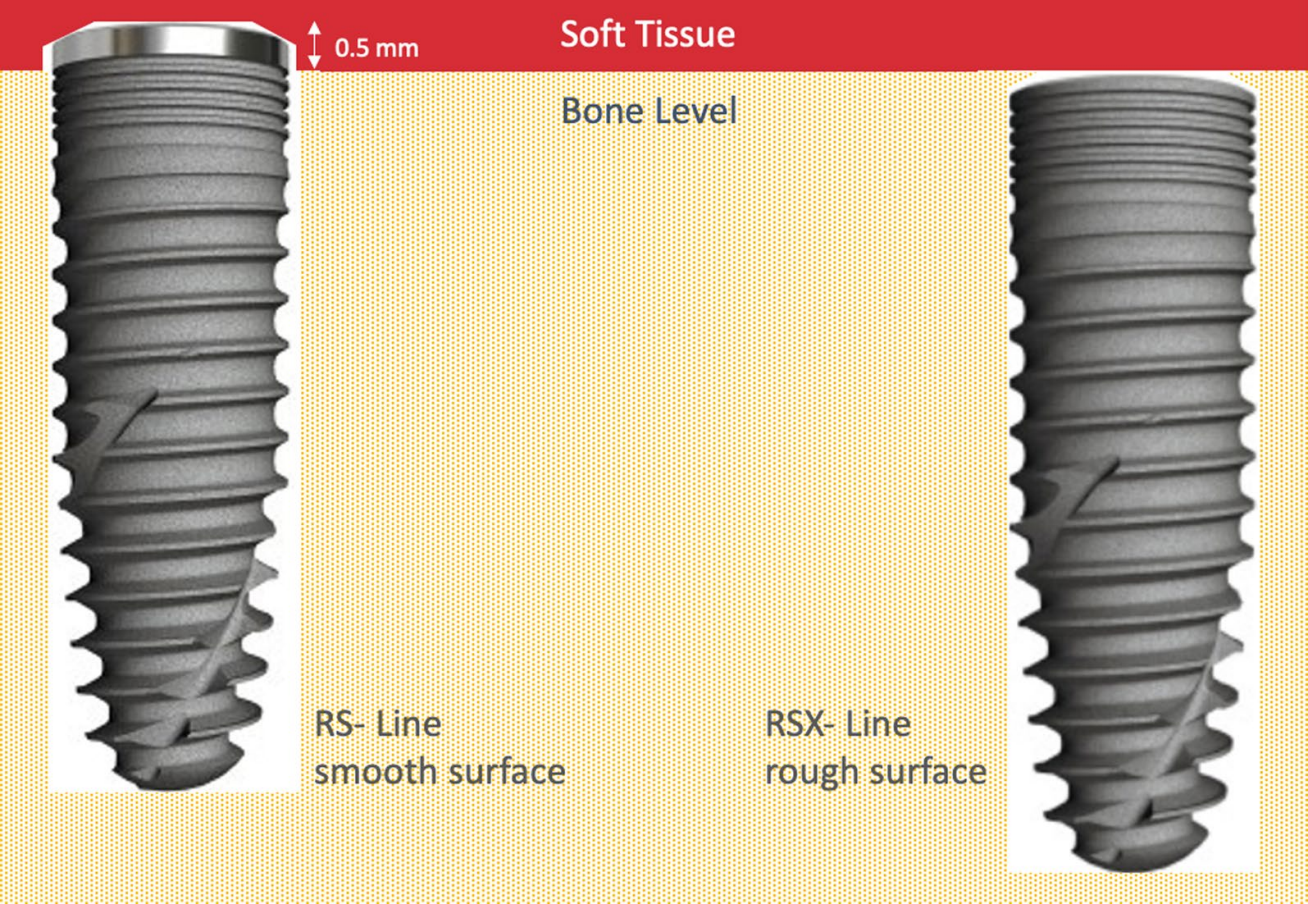
Implant macro-designs: RS-line implant left (polished shoulder) and RSX-line implant (rough shoulder) right. RSX-line implants were aimed to be completely inserted leveling the alveolar crest. The intended position for the RS-line implants was 0.5 mm supracrestal, thus positioning the polished shoulder in direct contact to the oral soft tissue environment

Two dental implants randomized for location (one RS-implant BEGO SEMADOS^®^ and one implant of the RSX-Line BEGO Semados^®^; BEGO Implant Systems GmbH & Co. KG, Bremen, Germany) were inserted in comparable regions of the lower or the upper jaw (adjacent or opposite), respectively. Randomization was performed using a standard randomization protocol (random.org, Randomness and Integrity Services Ltd, Dublin, Ireland) by the operating surgeon at site. Surgery was performed by highly experienced clinicians under local anesthesia. Both systems were inserted with the same surgical tray following working instructions recommended by the manufacturing company [17]. In accordance to the clinical standard procedure for implant placement, all RSX-line implants were aimed to be completely inserted leveling the alveolar crest. The intended position for the RS-line implants was 0.5 mm supracrestal, thus positioning the polished shoulder in direct contact to the oral soft tissue environment (Fig. 2). Prosthetic rehabilitation was performed only with fixed single crowns or fixed dental bridges according to the inclusion criteria.

### Implant description

Both implants hold the same features besides the surface characteristics of the implant shoulder (RS-line = machined versus RSX-line = rough/micro-structured) (Fig. 2). They are both made from commercially pure titanium (grade 4) with a high-purity and homogeneous TiPurePlus surface (sandblasted and etched). The surface roughness with Ra 1.6 µm is comparable to the roughness of a natural tooth root (Ra 1.7 μm) [18]. The macro-architectural shape is conical and self-tapping with a bionic thread and with a rounded apex to protect anatomical structures. The implant body is equipped with a self-tapping, bionic thread. They dispose a platform switch, an internal taper connection with a 45° medium taper angle and an internal hex anti-rotation protection. In the upper part of the implant body, the implants have bionic microgrooves. They are available in five different diameters (3.0, 3.75, 4.1, 4.5, 5.5 mm) and in six different lengths (7, 8.5, 10, 11.5, 13, 15 mm). The RS-line has a 0.5-mm machined (= polished) shoulder. The surface roughness in this part of the implant is about 0.4 µm. This value is close to the surface roughness of natural enamel, which lies between 0.07 and 0.5 µm [19]. The shoulder of the RSX-line is covered with the TiPurePlus surface and further structured with bionic microgrooves, thus the surface roughness in this region is equivalent to the remaining part of the implant (Ra 1.6 µm).

### Evaluation

**Fig. 3:**
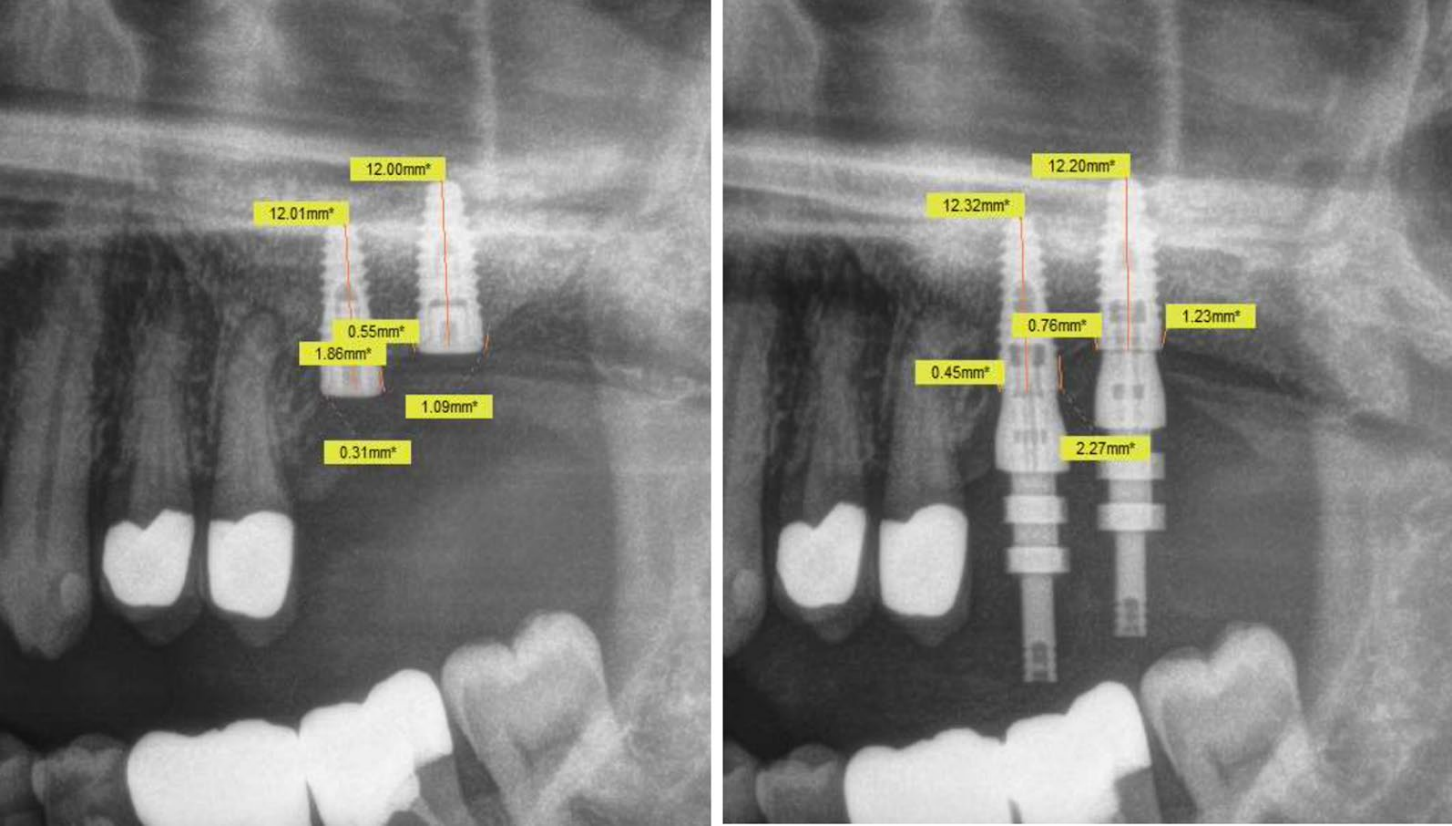
Analysis of radiographs using the measuring tool (Sidexis 4, Dentsply Sirona, York, USA) taken at the time of implantation (left) and after 6 months (right). The measuring tool was used to calculate distances between implant shoulder and marginal bone mesially and distally of the implant

All patients underwent clinical (Table 1) and radiographic examinations (digital panoramic imaging; Densply Sirona) after 6, 12 and 24 months (2014–2016). An independent, experienced, blinded examiner evaluated the data. Standardized case report forms were used for collecting the data. X-ray images were analyzed with the imaging processing software Sidexis 4 (Dentsply Sirona, York, USA) (Fig. 3) by always the same examiner. First, each radiograph was calibrated using a known reference distance, in this case the length of the implant. The distance between the implant shoulder and the point where the implant hits the alveolar bone first was measured mesial and distal of the implant. Application of the rule of three was used to obtain the distance. The height of the marginal bone level at implant insertion was used as base line marginal bone height. Taking base line marginal bone height into account, the effective marginal bone loss after 6, 12 and 24 months resulted in the difference between base line bone levels to the measured marginal bone levels during follow-up examination.

**Table 1:** Clinical criteria for peri-implant soft tissue health after 2 years including probing depth, bleeding on probing, recession, secretion, peri-implantitis and peri-implant mucositis separated for polished shoulder implants (RS-line implants), rough shoulder Implants (RSX-line implants) and both in the same patient

	RS-implant (polished)	RSX-implant (rough)	RS + RSX same patient	Rate in %
Probing depth 3–6 mm	12	10	8	19
Probing depth > 6 mm	1	1	1	1.7
Bleeding on probing (BOP)	9	10	8	16.3
Recession	4	4	4	6.9
Secretion	0	0	0	0
Peri-implantitis	1	1	1	1.7
Peri-impl. mucositis	9	10	8	16.3

### Statistical methods

Data were blinded for statistical analysis. For demographic data, clinical examination results and postsurgical treatment methods of descriptive statistics were used. Mean values and standard deviations (± SD) were calculated for the parameters implant insertion depth, bone level changes and probing depths. A Wilcoxon test was chosen to prove dependence relationship/to analyze the correlations between implant shoulder design and marginal bone loss. The *p*-value was set at 0.05. Data were analyzed for confounders (implant lengths, implant diameters, insertion site, augmentation, type of overdenture) by a regression analysis. Statistical analysis was performed using the statistical program IBM SPSS Statistics 22 and 28 (www.ibm.com).

## Results

### Demographic data

**Fig. 4:**
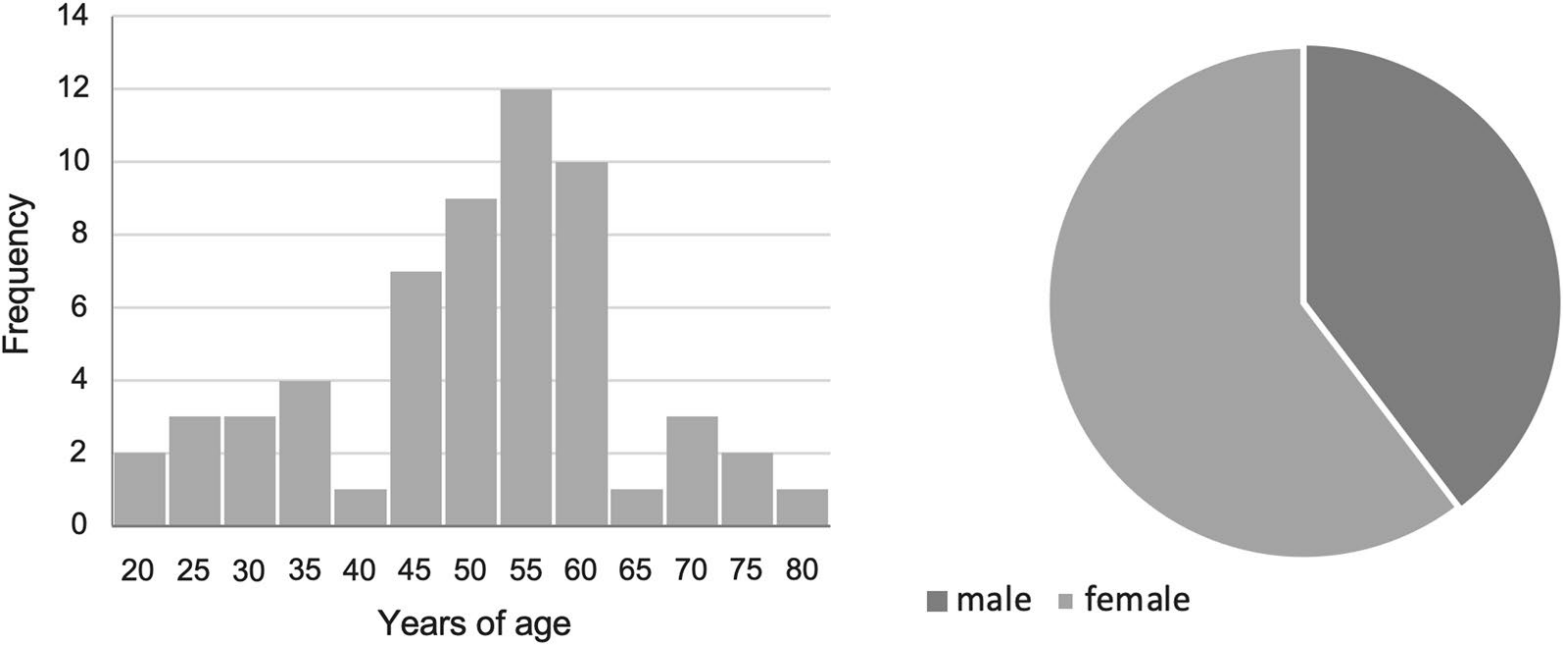
Age (right) and gender (left) distribution of participants (left)

Between 2012 and 2016, 58 patients aged 22 to 80 underwent implant therapy. Within this population there were 37.7% male and 60.3% female. Each patient received randomly one RS- and one RSX-implant in two regions of the jaw with similar bone quality (Fig. 4).

### Implant data

In the RS-implant group 31% of implants were 3.75 mm wide, 43.2% showed a diameter of 4.1 mm and 25% of 4.5 mm. Most implants in this group (48.3%) showed a length of 11.5 mm. Within the RSX group 27.6% of implants held a diameter of 3.75 mm, 50% 4.1 mm and 22.4% 4.5 mm. Equally to the RS group most implants were 11.5 mm long. No implants of 7 or 15 mm length and diameter of 3 mm or 5.5 mm were used. Therefore, in terms of implant sizes, the implant population showed a relatively homogenous distribution. In the RS group 13 patients and in the RSX group 11 patients received bone augmentation prior to implant insertion. All implants showed primary stability on manual testing at an insertion torque of 25 to 35 N cm.

Insertion depths were planned differently for both groups (RS = crestal; RSX: bone level), therefore crestal bone levels varied at insertion.

### Radiographic analysis

**Fig. 5:**
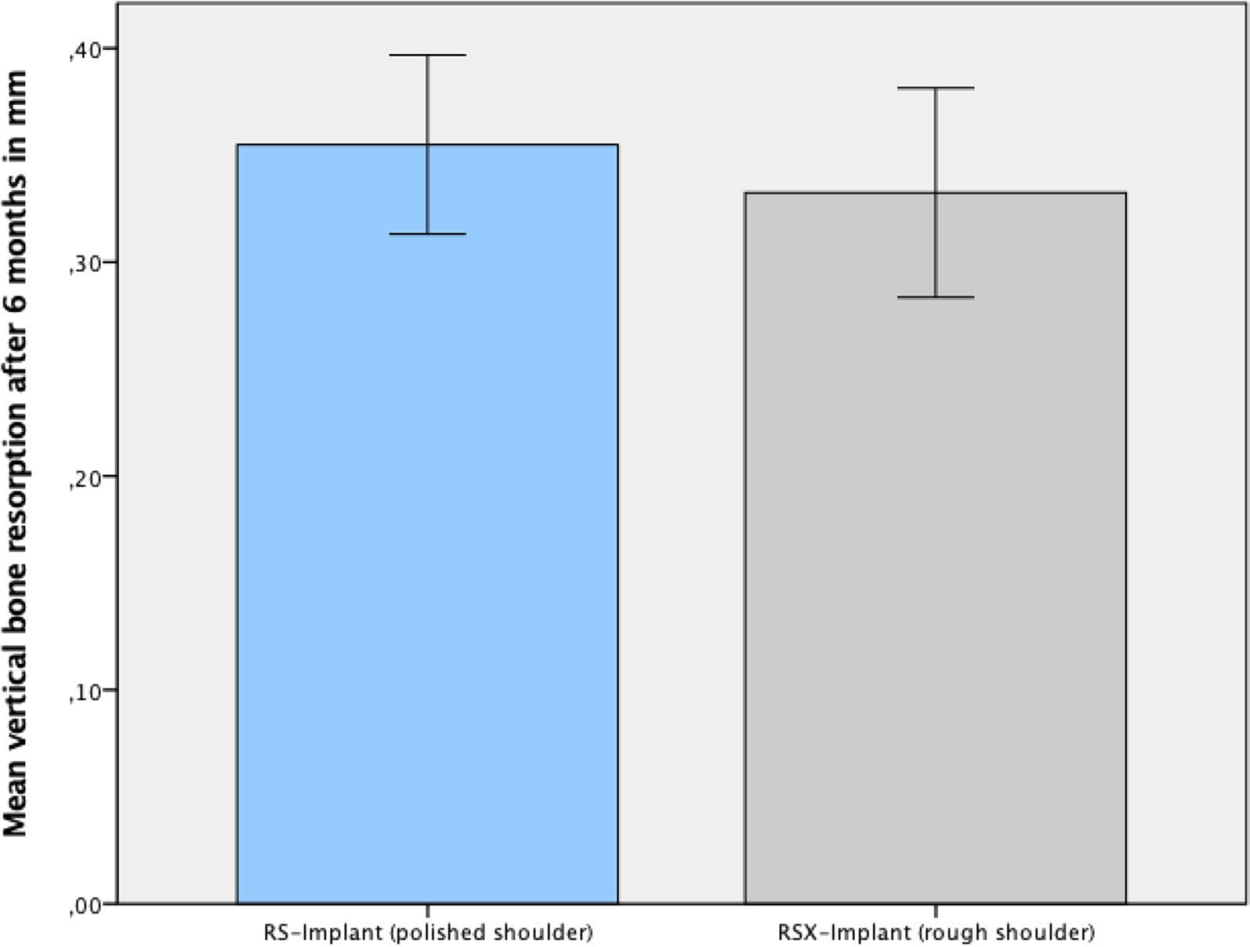
Average bone level changes in mm within 6 months left (polished shoulder) and RSX-line implant (rough shoulder) right

The radiographic analysis of bone levels directly after implant surgery showed an epicrestal position of 0.29 mm (SD ± 0.28) for the RS and 0.09 mm (SD ± 0.25 mm) for the RSX group. As expected, the two implant groups showed statistically significant differences in terms of base line bone levels (Wilcoxon test: *p* < 0.05) (Fig. 5).

Taking base line marginal bone level into account, the RS group showed an average bone level change of 0.35 mm (SD ± 0.19) and the RSX group of 0.32 mm (SD ± 0.22) within 6 months of healing. The statistical analysis showed no difference in bone resorption between both groups (Fig. 5, Table 2). One year after implant placement bone was resorbed on average by 0.81 mm in the RS group and by 0.62 mm in the RSX group. Bone level changes were 0.52 mm (SD ± 0.36 mm) in the RS group and 0.53 mm (SD ± 0.32 mm) in the RSX group, respectively (Table 2). No statistic difference in bone level changes was observed between both groups.

**Table 2:** Marginal bone level changes 6 months, 1 year and 2 years after surgery in mm

Time of measurement	Implant group	N	Min	Max	Mean	SD
6 months	RS (polished)	58	0.10	0.70	0.35	0.19
	RSX (rough)	58	0.00	0.70	0.32	0.22
1 year	RS (polished)	58	0.00	1.50	0.52	0.36
	RSX (rough)	58	0.10	1.30	0.53	0.32
2 years	RS (polished)	58	0.20	1.40	0.61	0.28
	RSX (rough)	58	0.20	1.15	0.58	0.24

Mean marginal bone levels differ only slightly in between the two groups

**Fig. 6:**
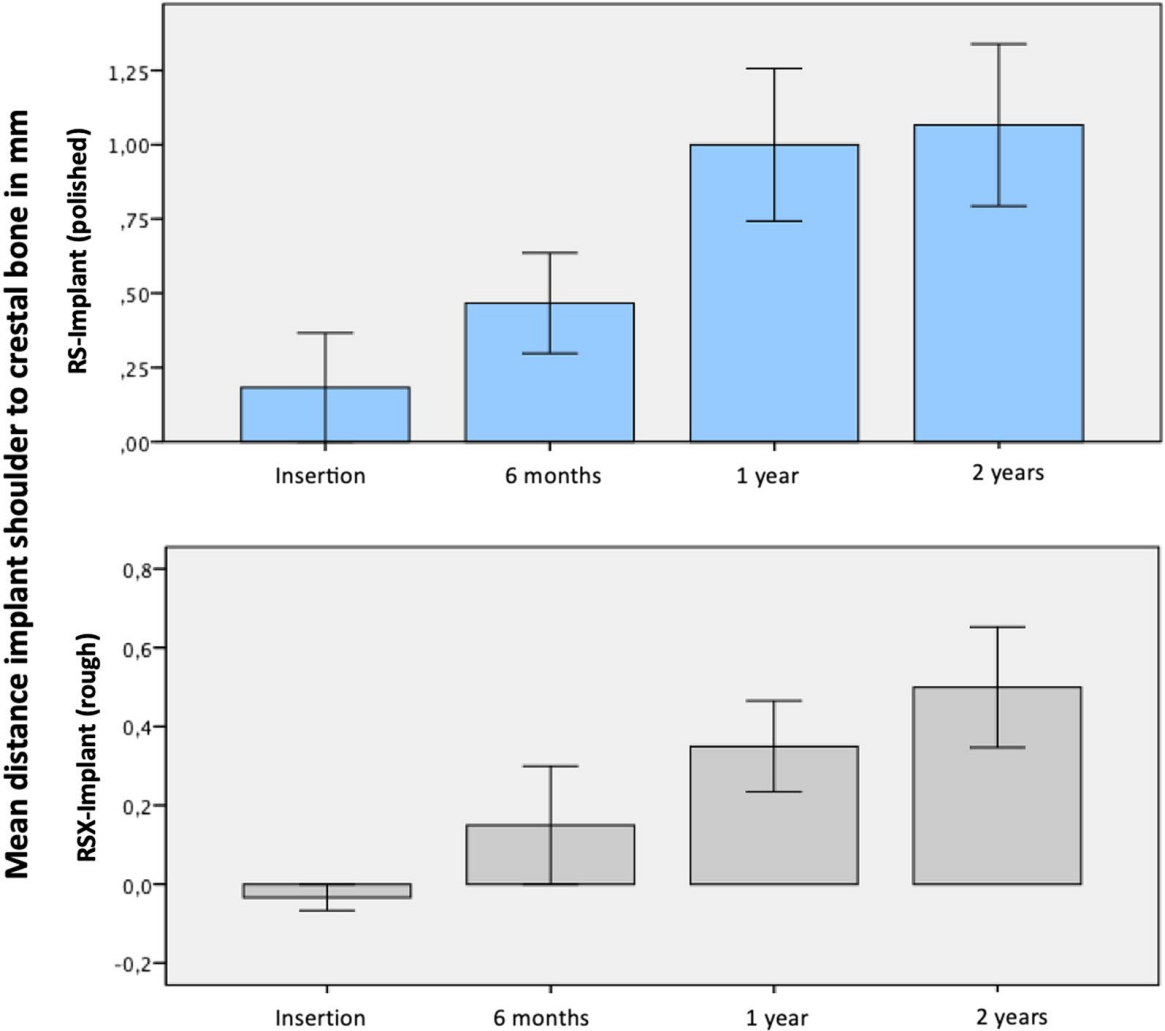
Distance from implant shoulder to the first bone-to-implant contact in both groups

Moreover, no statistically significant differences in bone loss occurred after 2 years between both implant groups. The RS implants showed an average bone loss of 0.61 mm (SD ± 0.28 mm), the RSX implants showed 0.58 mm of average bone loss (SD ± 0.24 mm) (Table 2). Similar bone level changes were observed in all implants. As shown in Table 2, bone level changes occurred parallel to each other. There were no statistically significant differences in effective bone level changes. Due to different insertion depth bone level heights varied for both implants (RS and RSX) (Figs. 5, 6).

### Clinical evaluation

Overall, no implant was lost within the 2 years of follow-up, revealing a survival rate of 100%. There were no significant differences in terms of peri-implant soft tissue health in the period from surgery to 2-year control. Only one RS and one RSX implant had an average probing depth > 6 mm (both in the same patient). Eleven patients had indications for a peri-implant mucositis. Bleeding on probing in combination with a probing depth of 3–6 mm was seen in 7.6% in the RS and in 8.3% in the RSX group. Recession was observed in four patients in both implants. Secretion or implant mobility was not observed within study time.

## Discussion

This prospective randomized-controlled clinical study was conducted to evaluate the impact of implant shoulder design on marginal bone resorption. No significant difference in the influence of machined or micro-structured implant collar region on peri-implant crestal bone levels or on peri-implant soft tissue health could be found. The difference in marginal bone resorption of 0.04 mm was statistically not significant (*p* = 0.7; Wilcoxon test).

Comparable results were observed in literature. Bassetti et al. and Karlsson et al. described that implants with machined and implants with rough neck design both fulfilled implant success criteria and that the modification in implant neck texture had no significant influence on peri-implant bone, resulting in the same amount of bone loss [20, 21]. Other authors, accordingly, could not find a significant association between a special implant design or implant surface on crestal bone changes [22, 23]. Furthermore, no implants characteristics were found to be related to the presence of peri-implantitis [24, 25].

The average peri-implant bone resorption in the present study was in accordance with current literature. The RS implants showed a mean bone resorption of 0.61 mm (SD ± 0.28 mm) and the RSX implants 0.57 mm (SD ± 0.24 mm) 2 years after implant insertion. Other studies showed marginal bone loss of 1.07 mm after 10 years [26] and 0.36 mm (SD; 0.55) after 2 years [27]. Buser et al. detected radiologically a mean distance from the implant shoulder to the first bone-to-implant contact of 3.32 mm (SD ± 0.73) 10 years after surgery [28]. Nicolau et al. noticed a bone loss of max. 2.00 mm ± 1.19 mm within 10 years [29]. After an initial remodeling phase (5 to 6 months) where the depth of implant placement had an influence on the initial bone remodeling, no significant differences were detected [29]. Several studies report bone resorption up to 1.5/2 mm during the 1st year after insertion as a normal remodeling process and consider a further marginal bone loss of 0–0.2 mm in following years as a physiological progression [30–33].

Recent studies defined the impact of platform switching [34] and the effect of bone loading [35] as critical factors in maintaining peri-implant bone levels. Today’s literature on implant shoulder design and the effect on marginal bone remodeling is still very inconsistent. Some authors describe a rather positive influence using roughed neck implants. Koodaryan et al. showed in their systematic review including 12 articles that insertion of implants with rough and rough-surfaced micro-threaded neck design influenced the rate of bone loss and favored lesser marginal bone resorption compared to machined-neck implants [30].

In contrast, Sánchez-Siles et al. observed in their retrospective study including 1244 implants that the amount of marginal bone loss was significantly lower in smooth-necked implants (1.18 ± 1.39 mm) compared to rough-surfaced implants (2.41 ± 1.35 mm) after 10 years of function (*p* < 0.001) [36]. Valderrama et al. even reported a mean bone gain of 0.11 mm for non-machined collar implants compared to a mean bone loss of 1.00 mm for implants with a machined collar of 2.8 mm [37]. A review in 2018 found more crestal bone resorption when rough collars were used in comparison to the use of machined [38].

Comparing implants with differently wide machined collar surfaces also led to unequal conclusions in literature [25]. Hänggi et al. on one hand observed that bone remodeling did not significantly differ between two types of implants with differently wide machined-neck implants (2.8 mm vs. 1.8 mm) [39]. While on the other hand a histomorphometric analysis performed by Schwarz et al. obtained lower mean implant shoulder-to-crestal bone contact values for implants with shorter/thinner machined neck; 0.4 mm vs. 1.6 mm [16]. Similar findings were described by Calvo-Guirado et al. [15]. The machined collar neck of the RS implant used in the present study, with a width of 0.5 mm, therefore lies in the recommended dimension.

Radiographic evaluation is the most common method in literature to assess bone loss. Unfortunately, conventional radiographs only monitor the mesial or distal aspect of bone loss around the implant body [40]. There are in general limitations of two-dimensional radiological measurements provided in most studies [41]. In this study in particular, as panoramic images were used for follow-ups. Periapical radiographs should have been used. Those have proven reliable in determining the bone level changes at different follow-ups [42].

According to the manufacturer’s instructions and to clinical and scientific proposals the machined-neck implants were inserted positioning the machined neck 0.29 mm (SD ± 0.28) above the crestal bone. Although the non-machined neck implants were proposed to be inserted equi- or subcrestal, they ended up in a slightly epicrestal position with an average value of 0.09 mm (SD ± 0.25 mm). Nevertheless, there was a statistically significant difference in insertion depths as expected (Wilcoxon test: *p* < 0.05). These different insertion depths limit results of the study’s scientific significance of the comparison especially in light of the short follow-up period of only 2 years. Schwarz et al. described in his review including 13 publications different outcomes of crestal bone changes after insertion of implants with the same design in different positions relative to the crestal bone [43]. It remains questionable, if the effect of implants with different designed collars and additionally various insertion depth are comparable. Furthermore, although data were assessed for possible confounders (implant lengths and diameter, insertion site, augmentation, type of overdenture) other not evaluated factors such as angulation, three different surgeons preforming the operations, patient-specific factors, one examiner measuring the bone loss, and the type of examination (measurement methods) might limit conclusions of this study.

Hüzeler et al. described a statistical difference for base-line mean values of crestal bone height and for mean bone level changes after 1 year comparing peri-implant bone levels around implants with and without platform switching. The concept of a platform switching appeared to limit crestal resorption [32]. Also, other studies showed a relationship between reduced marginal bone level changes and the presence of platform switching [44]. All implants used in the present study are equipped with platform switching. There is evidence that the characteristics of a platform switch are favorable to keep biological width and therefore reduce marginal bone resorption [45].

In the presented work, there were no differences in evaluation of peri-implant soft tissue health between machined and non-machined neck implants. One patient developed a peri-implantitis in both inserted implants with probing depth higher than 6 mm and bleeding on probing. This peri-implantitis rate is in correlation with the true “peri-implantitis” frequency of 1–2% suggested by Albrektsson et al. during a follow-up period of 10 years or more [46].

It is suggested that peri-implantitis progresses in a non-linear, accelerating pattern and that, for the majority of cases, the onset occurs early during follow-up within 3 years of function [47, 48]. Besides, peri-implantitis is considered to be directly linked to surface roughness [11]. The surface roughness of the machined collar of implants used in this study was with a value of 0.4 µm within the recommended range. This value also lies within the surface roughness of natural enamel which is reported to be between 0.07–0.5 µm [19, 49, 50].

However, most studies on the impact of implant collar design on marginal bone levels are performed in animal studies [41]. There is a lack of evidence from clinical trials reflecting real-life situations, as animal models may not completely recreate the anatomical, physiological, biochemical/functional, or pathological environment of clinical conditions in humans [51]. According to the present body of literature, there is no implant surface or material, reducing the risk for peri-implantitis [25]. In terms of biofilm adsorption recent studies showed that roughness does not influence the diversity of the microbiome [52].

Furthermore, it was observed that several studies in literature did not consider the initial position of the implant neck relative to the crest, resulting in distorted values of mean bone losses [41]. This fact, besides others, makes it very difficult to compare any study evaluating peri-implant bone changes.

## Conclusions

The aim of this study was to investigate the influence of the architecture of the collar region on marginal bone and soft tissue response. No significant differences on crestal bone loss and on soft tissue response were observed between implants with machined and roughened neck on marginal bone. None of both collar designs showed a clear advantage in peri-implant reaction. Both implant systems achieved equally good results concerning peri-implant bone loss, the rate of peri-implantitis and hard and soft tissue integration. Nevertheless, more evidence is needed on how to design the collar region, where the implant is in close contact to the oral cavity. Further investigation on this topic should be performed with higher patient numbers to find the best outcome for osseointegration, simultaneously reducing peri-implant inflammation to a minimum.

## Supplementary Information

**Additional file 1.** Implant positions, implant diameters and implant lengths of included patients.

## Data Availability

The datasets used and/or analyzed during the study are available from the corresponding author upon reasonable request.
