# Supplementary material for: Impact of machined versus structured implant shoulder designs on crestal bone level changes: a randomized, controlled, multicenter study
Source: Int J Implant Dent. 2022 Jul 16;8:31. doi: 10.1186/s40729-022-00432-4 (PMC9288572; doi:10.1186/s40729-022-00432-4)
Supplement: Supplementary file 1 — Additional file 1. Implant positions, implant diameters and implant lengths of included patients. [file 40729_2022_432_MOESM1_ESM.docx]

| **Patient number** | **RS-implant** | | | **RSX- implant** | | |
| --- | --- | --- | --- | --- | --- | --- |
|  | **position** | **diameter in mm** | **length**  **in mm** | **position** | **diameter in mm** | **length in mm** |
| 1 | 35 | 3.75 | 13 | 45 | 3.75 | 13 |
| 2 | 45 | 3.75 | 10 | 35 | 3.75 | 10 |
| 3 | 16 | 4.1 | 11.5 | 26 | 4.1 | 11.5 |
| 4 | 36 | 3.75 | 10 | 46 | 3.75 | 10 |
| 5 | 36 | 4.1 | 11.5 | 46 | 4.1 | 11.5 |
| 6 | 36 | 3.75 | 11.5 | 46 | 3.75 | 11.5 |
| 7 | 16 | 4.1 | 11.5 | 26 | 4.1 | 11.5 |
| 8 | 46 | 4.5 | 11.5 | 36 | 4.5 | 11.5 |
| 9 | 45 | 3.75 | 11.5 | 35 | 3.75 | 11.5 |
| 10 | 46 | 4.5 | 11.5 | 36 | 4.5 | 11.5 |
| 11 | 16 | 4.5 | 11.5 | 37 | 4.5 | 11.5 |
| 12 | 15 | 3.75 | 11.5 | 25 | 3.75 | 11.5 |
| 13 | 17 | 4.1 | 11.5 | 27 | 4.1 | 11.5 |
| 14 | 46 | 3.75 | 13 | 36 | 3.75 | 13 |
| 15 | 17 | 4.1 | 11.5 | 16 | 4.1 | 11.5 |
| 16 | 21 | 3.75 | 11.5 | 11 | 3.75 | 11.5 |
| 17 | 26 | 4.1 | 10 | 27 | 4.5 | 10 |
| 18 | 26 | 4.5 | 11.5 | 25 | 4.5 | 10 |
| 19 | 26 | 4.5 | 13 | 25 | 4.1 | 13 |
| 20 | 17 | 4.5 | 10 | 46 | 4.5 | 11.5 |
| 21 | 44 | 3.75 | 11.5 | 46 | 3.75 | 10 |
| 22 | 16 | 4.5 | 13 | 25 | 4.5 | 13 |
| 23 | 26 | 4.5 | 13 | 24 | 4.1 | 11.5 |
| 24 | 46 | 4.1 | 10 | 47 | 4.1 | 10 |
| 25 | 46 | 4.1 | 11.5 | 47 | 4.1 | 8.5 |
| 26 | 37 | 4.5 | 10 | 36 | 4.1 | 11.5 |
| 27 | 27 | 4.1 | 8.5 | 26 | 4.1 | 8.5 |
| 28 | 26 | 4.5 | 13 | 27 | 4.5 | 13 |
| 29 | 11 | 4.1 | 13 | 12 | 4.1 | 13 |
| 30 | 34 | 3.75 | 11.5 | 36 | 3.75 | 11.5 |
| 31 | 26 | 4.1 | 10 | 24 | 4.1 | 13 |
| 32 | 37 | 3.75 | 8.5 | 36 | 3.75 | 8.5 |
| 33 | 16 | 4.1 | 10 | 14 | 4.1 | 10 |
| 34 | 45 | 3.75 | 10 | 35 | 3.75 | 10 |
| 35 | 17 | 4.5 | 10 | 16 | 4.1 | 10 |
| 36 | 16 | 4.1 | 10 | 15 | 4.1 | 10 |
| 37 | 24 | 3.75 | 11.5 | 25 | 3.75 | 11.5 |
| 38 | 14 | 4.1 | 13 | 13 | 4.1 | 11.5 |
| 39 | 16 | 4.1 | 11.5 | 15 | 4.1 | 11.5 |
| 40 | 34 | 4.1 | 11.5 | 44 | 4.1 | 11.5 |
| 41 | 16 | 4.1 | 11.5 | 17 | 4.1 | 11.5 |
| 42 | 35 | 4.5 | 10 | 37 | 4.5 | 8.5 |
| 43 | 47 | 4.1 | 10 | 46 | 4.1 | 10 |
| 44 | 25 | 4.1 | 13 | 15 | 4.1 | 13 |
| 45 | 22 | 3.75 | 11.5 | 12 | 3.75 | 11.5 |
| 46 | 26 | 4.5 | 11.5 | 16 | 4.5 | 11.5 |
| 47 | 13 | 4.1 | 13 | 23 | 4.1 | 13 |
| 48 | 45 | 3.75 | 8.5 | 46 | 3.75 | 10 |
| 49 | 16 | 4.5 | 13 | 17 | 4.5 | 13 |
| 50 | 35 | 3.75 | 8.5 | 36 | 3.75 | 10 |
| 51 | 16 | 4.5 | 13 | 26 | 4.5 | 13 |
| 52 | 46 | 3.75 | 11.5 | 45 | 4.1 | 10 |
| 53 | 42 | 4.1 | 10 | 32 | 4.1 | 10 |
| 54 | 26 | 4.1 | 11.5 | 16 | 4.1 | 11.5 |
| 55 | 46 | 4.1 | 11.5 | 47 | 4.1 | 11.5 |
| 56 | 37 | 3.75 | 11.5 | 26 | 4.5 | 11.5 |
| 57 | 25 | 4.1 | 11.5 | 26 | 4.1 | 11.5 |
| 58 | 35 | 4.1 | 11.5 | 36 | 4.1 | 11.5 |

Table 3: Implant positions. Implant diameters and Implant lengths of included patients.
